# Chimeric Free Fibula Flap: An Encounter With Aberrant Anatomy

**DOI:** 10.7759/cureus.10073

**Published:** 2020-08-27

**Authors:** Dieter Brummund, Angela Chang

**Affiliations:** 1 Department of General Surgery, Aventura Hospital and Medical Center, Aventura, USA; 2 Department of Anesthesia, Aventura Hospital and Medical Center, Aventura, USA

**Keywords:** free fibula flap, free tissue transfer, osteocutaneous flap, septocutaneous perforator, myocutaneous perforator, oral cancer, anastomosis, flap salvage

## Abstract

A 59-year-old male presented with recurrent mucoepidermoid carcinoma of the mandible. A resection with immediate free fibula flap reconstruction was done. The osteocutaneous free fibula flap relies on the peroneal artery and its distal perforators. Variant patterns necessitate consideration of the challenging to dissect proximal myocutaneous perforator raised on a single or double anastomosis, depending on origin. Even in cases of flap salvage, the fibula flap remains a reliable flap. This case describes a fibula flap with a sole proximal myocutaneous perforator identified during dissection despite a normal preoperative Doppler.

## Introduction

The flap of choice for mandibular reconstruction is the osteocutaneous free fibula flap, which is based on the peroneal artery and its perforating vessels. Lykoudis et al. found four to seven perforators greater than 0.5 mm in diameter per leg, of which 70% were septocutaneous, 14.2% septomusculocutaneous, and 15.6% musculocutaneous. Septocutaneous perforators are reliably found distally and myocutaneous perforators more proximally. The proximal perforators typically follow a long oblique course and distal perforators travel a short transverse course. The preferred perforator pattern for osteocutaneous flap dissection is septocutaneous, given the distal location and ease of dissection through the avascular plane of the posterior intermuscular septum through which they travel [1].

The peroneal artery, upon which the fibula flap is based, is a remnant of the embryologic sciatic artery. During development, the distal extremity and foot are initially supplied by the sciatic artery. As development continues, the sciatic artery regresses and the femoral artery becomes dominant. Interindividual variability in the degree of regression and anastomosis between the two results in variant vascular patterns [2]. Variant terminal branching of the popliteal artery occurs at a rate of approximately 10% in anatomic studies, with the three most common branding patterns being popliteal trifurcation, anterior tibioperoneal trunk, and high terminal division [3]. While these variants exist, preoperative angiography is generally only warranted in patients with a history of lower extremity trauma and or abnormal clinical examination of pulses and Doppler signals of the extremity [4].

## Case presentation

**Figure 1:**
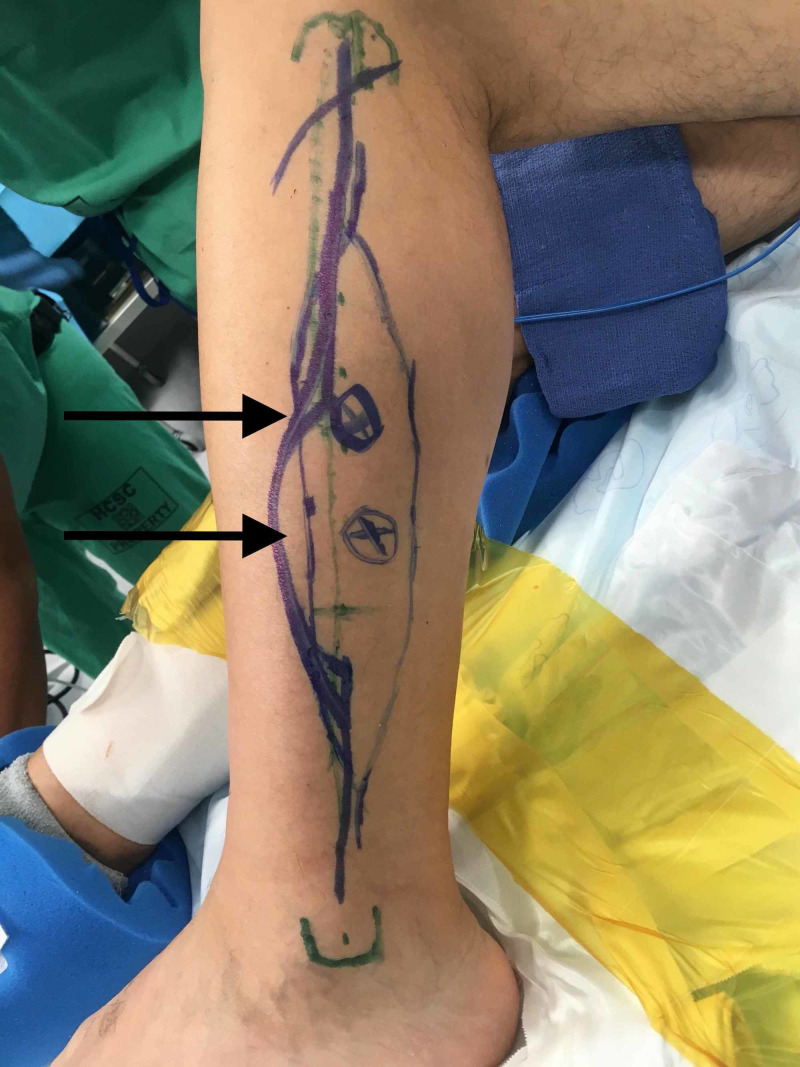
Preoperative fibula flap markings including dopplerable perforators (arrows)

A 59-year-old male presented with recurrent mucoepidermoid carcinoma of the mandible. Preoperative ultrasound identified two perforators overlying the middle third of the fibula (Figure 1).

**Figure 2:**
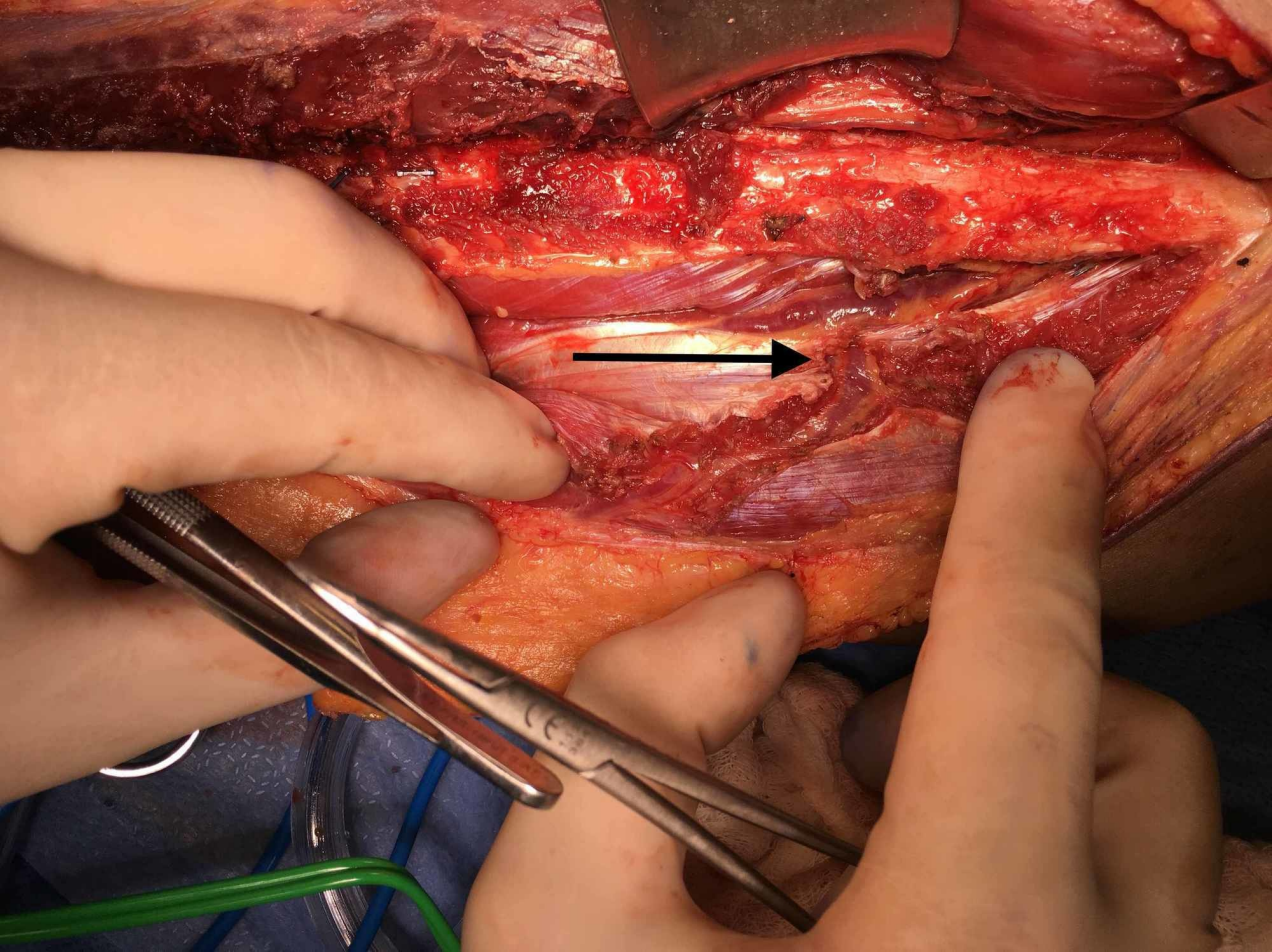
Mid-dissection myocutaneous perforator identification (arrow)

Resection with immediate free fibula flap reconstruction of the defect was performed. Distal dissection of the fibula flap along the posterior intermuscular septum found no septocutaneous perforators. Proximally, a single myocutaneous perforator was identified originating from the peroneal artery just distal to the tibioperoneal bifurcation (Figure 2).

**Figure 3:**
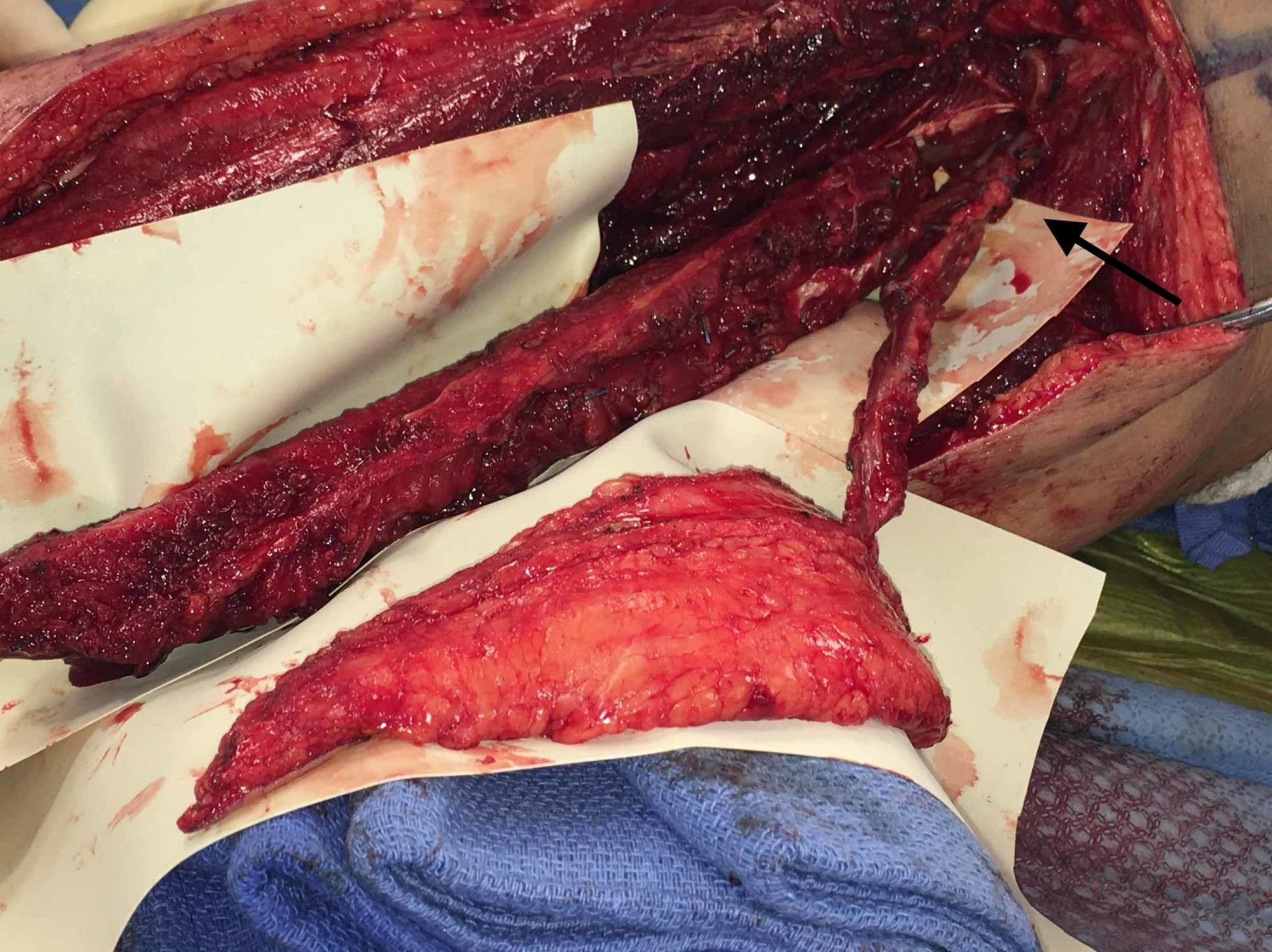
Dissected chimeric fibula flap with short common peroneal artery pedicle (arrow)

The flap was raised as a chimeric flap with separate skin and osseous components (Figure 3) and secured to the defect area. Postoperative monitoring was done using In Vivo Optical Spectroscopy (INVOS^TM^, Medtronic, Minneapolis, MN, USA). INVOS is a near-infrared oximetry monitoring device allowing for real time monitoring of flap perfusion [5]. The patient suffered no significant postoperative complications and was discharged home on postoperative day 5. 

## Discussion

Congenital skin perforator variants follow a convergent or divergent pattern according to their relation to the peroneal artery. A convergent perforator system arises directly from the peroneal artery in 50% of flaps and can be raised as a single composite unit. A divergent perforator arises from a vessel other than the peroneal artery, from the posterior tibial artery in 35% of flaps or the tibioperoneal trunk in 5%. These patterns require dissection of the flap as two separate components. Daya describes a convergent system raised as a double skin paddle chimeric flap for additional soft tissue coverage in a complex oromandibular reconstruction following a shotgun injury [6]. Divergent systems have been salvaged by intraflap anastomoses in series, or by parallel anastomosis of each component at the recipient site [7]. Alternative salvage strategies when encountering atypical vascularity include separating the skin paddle from the osseous component and raising both flaps individually, harvesting a separate cutaneous flap, a dual skin component design, using the contralateral leg, or abandoning the flap all together. It is important to note that even when flap salvage must occur, the fibula is a viable flap with a low incidence of flap loss [8,9].

We reviewed the literature for similar cases to our fibula flap with no septocutaneous perforator that relied on a single myocutaneous proximal peroneal artery perforator. Winters et al. reports an incidence of a single myo- or septomyocutaneous peroneal perforator at 10% in a series of 20 cases [9]. Wong et al. reports an incidence of no septocutaneous perforator at 3% in a series of 1,100 patients [10]. Taking both series into consideration, we estimate a combined incidence of 0.3% for the vascular pattern we encountered.

## Conclusions

This case report describes a free fibula flap with no septocutaneous perforator. The flap was salvaged by carefully extending the dissection proximally until a myocutaneous perforator was found. Then it was raised as a chimeric flap with skin and osseous components. This case highlights that proximal myocutaneous perforators should always be preserved until the presence of a septocutaneous perforator is confirmed to allow for flap salvage in the rare, but real, possibility of aberrant vasculature.
